# Mutation of *SALL2* causes recessive ocular coloboma in humans and mice

**DOI:** 10.1093/hmg/ddt643

**Published:** 2014-01-09

**Authors:** Daniel Kelberman, Lily Islam, Jörn Lakowski, Chiara Bacchelli, Estelle Chanudet, Francesco Lescai, Aara Patel, Elia Stupka, Anja Buck, Stephan Wolf, Philip L. Beales, Thomas S. Jacques, Maria Bitner-Glindzicz, Alki Liasis, Ordan J. Lehmann, Jürgen Kohlhase, Ken K. Nischal, Jane C. Sowden

**Affiliations:** 1 Ulverscroft Vision Research Group; 2 Developmental Biology Unit, Birth Defects Research Centre; 3 Clinical and Molecular Genetics Unit; 4 Centre for Translational Genomics – GOSgene and; 5 Neural Development Unit, UCL Institute of Child Health, 30 Guilford Street, London WC1N 1EH, UK; 6 Institute for Human Genetics, University of Goettingen, Germany; 7 Department of Histopathology and; 8 Clinical and Academic Department of Ophthalmology, Great Ormond Street Hospital for Children NHS Foundation Trust, London WC1N 3JH, UK; 9 Department of Ophthalmology and Medical Genetics, University of Alberta, Edmonton, CanadaT6G 2H7; 10 Center for Human Genetics Freiburg, Heinrich-von-Stephan-Str. 5, 79100 Freiburg, Germany; 11 UPMC Childrens Hospital of Pittsburgh and Eye Center, 4401 Penn Avenue, Pittsburgh, PA 15224, USA

## Abstract

Ocular coloboma is a congenital defect resulting from failure of normal closure of the optic fissure during embryonic eye development. This birth defect causes childhood blindness worldwide, yet the genetic etiology is poorly understood. Here, we identified a novel homozygous mutation in the *SALL2* gene in members of a consanguineous family affected with non-syndromic ocular coloboma variably affecting the iris and retina. This mutation, c.85G>T, introduces a premature termination codon (p.Glu29*) predicted to truncate the SALL2 protein so that it lacks three clusters of zinc-finger motifs that are essential for DNA-binding activity. This discovery identifies *SALL2* as the third member of the *Drosophila* homeotic *Spalt-*like family of developmental transcription factor genes implicated in human disease. *SALL2* is expressed in the developing human retina at the time of, and subsequent to, optic fissure closure. Analysis of *Sall2*-deficient mouse embryos revealed delayed apposition of the optic fissure margins and the persistence of an anterior retinal coloboma phenotype after birth. *Sall2*-deficient embryos displayed correct posterior closure toward the optic nerve head, and upon contact of the fissure margins, dissolution of the basal lamina occurred and PAX2, known to be critical for this process, was expressed normally. Anterior closure was disrupted with the fissure margins failing to meet, or in some cases misaligning leading to a retinal lesion. These observations demonstrate, for the first time, a role for *SALL2* in eye morphogenesis and that loss of function of the gene causes ocular coloboma in humans and mice.

## INTRODUCTION

Coloboma is an ocular birth defect resulting from abnormal development of the eye during embryogenesis. It represents an important cause of congenital blindness and visual impairment, estimated to account for 3–11% of blindness in children worldwide (1). The prevalence varies by population ranging from 4 to 19 per 100 000 live births across Europe (2–6) with higher rates reported in populations with high degrees of consanguinity (6,7). Coloboma is defined as a congenital defect in any ocular tissue(s), typically presenting as absent tissue or a gap, at a site consistent with aberrant closure of the optic fissure (1). The embryonic optic fissure is a transient ventral opening that arises during invagination of the optic vesicle in the formation of the bilayered optic cup. It permits the migration of periocular mesenchymal cells (mostly of neural crest origin) into the developing eye to form the hyaloid artery (1). During subsequent growth of the optic cup, the edges of the fissure align and fuse completing formation of the eye globe so that the ventral and dorsal retina become morphologically indistinguishable. In human development, the fissure narrows and begins to close during the 5th week, starting centrally and progressing anteriorly and posteriorly, and is normally completely fused by the end of the 7th week (1,8). Failure of fusion can lead to coloboma of one or multiple regions of the inferior portion of the eye affecting any part of the globe traversed by the fissure, from the iris to the optic nerve including the ciliary body, retina and choroid (9). Coloboma is also frequently associated with small (microphthalmic) or absent (anophthalmic) eyes (2,5) as part of an interrelated spectrum of developmental eye anomalies, and can affect either one or both eyes.

The molecular pathways controlling the events involved in closure of the optic fissure are largely unknown. Current knowledge of the mechanisms of tissue fusion throughout development are limited and are of considerable interest as failure of tissue fusion also accounts for other common human birth defects such as cleft palate and neural tube defects (10,11). Clinical, epidemiological and experimental evidence from animal models indicate a strong genetic basis. Recurrence risks for siblings of patients affected with optic fissure closure defects have been suggested to be between 8 and 13% (3). However, a recent UK study estimated that ∼40% of coloboma cases were likely to have a genetic cause based on positive family history or suspected familial syndrome (12). Genes associated with coloboma phenotypes have been identified in at least 20 syndromes, where the coloboma arises as an occasional feature of a complex multisystem birth defect (9,13–17); these include dominant mutations in *SHH* (OMIM 600725) (18), *RAX* (OMIM 601881) (19), *GDF3* (OMIM 606522) and *GDF6* (OMIM 601147) (13,20) and recessive mutations in *STRA6* (OMIM 610745) (21) and *SMOC1* (OMIM 608488) (22). Additionally, rare cases of non-syndromic coloboma have also been identified in patients with recessive mutations of *VSX2* (formerly *CHX10*; OMIM 142993) (23) and *ABCB6* (OMIM 605452) (24) more frequently associated with microphthalmia, dominant mutations of *PAX6* (OMIM 607108) generally associated with a range of ocular defects including aniridia, and *MAF* (OMIM 610210) (cataract and anterior segment dysgenesis) (23–26). The most commonly identified genetic causes of isolated coloboma, without microphthalmia, are *CHD7* (OMIM 608892) mutations associated with CHARGE syndrome (OMIM 214800) and *PAX2* mutations, which cause renal-coloboma syndrome (OMIM 167409) (12,27,28). However, in the majority of cases, the genetic contribution to ocular coloboma phenotypes remains to be determined (9,13,14,16,20). The findings to date indicate significant genetic heterogeneity and suggest perturbation at multiple stages of eye development can result in failure of optic fissure closure. A comprehensive understanding of pathogenic genetic changes is required to resolve the molecular etiology of coloboma.

Here, we report the identification of a novel homozygous mutation in the *SALL2* gene using a combined strategy of homozygosity mapping and exome sequencing in three siblings from a consanguineous family with an iris and retinochoroidal coloboma phenotype. This mutation introduces a premature stop codon predicted to result in loss of function of the mutant protein. RNA *in situ* hybridization of human embryonic eyes revealed expression of *SALL2* transcripts within the developing retina at the time of, and subsequent to, closure of the optic fissure (between 5 and 7 weeks of development). Furthermore, we show that mice with homozygous loss of the orthologous *Sall2* gene exhibit an abnormal closure process and a variable ocular coloboma phenotype, implicating a conserved role for the gene in eye morphogenesis, and specifically optic fissure closure, in humans and mice.

## RESULTS

### Patient phenotypes

**Figure 1.**
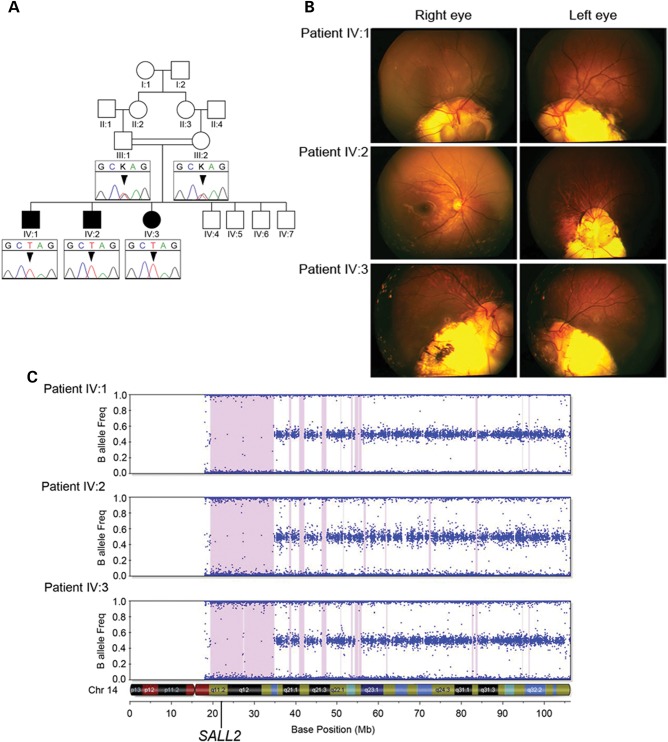
Pedigree structure, clinical images and homozygosity mapping. (**A**) Simplified pedigree of the family with electropherograms of part of *SALL2* exon 2 showing the c.85G>T mutation (arrowhead) homozygous in all three affected children and heterozygous in their parents. (**B**) Fundus photographs from all three affected children showing varying degrees of retinochoroidal coloboma affecting the optic disc in all cases except the right eye of patient IV:2 which has a normal appearance. (**C**) Homozygosity mapping output for each of the three affected siblings from Illumina Beadstudio v3.2 for chromosome 14 containing *SALL2*. Each dot represents an individual SNP marker on the array plotted as a function of the frequency of the minor (B) allele. Regions of extended homozygosity (>100 SNPs) are indicated by pink shading.

Three siblings born of first-cousin parents of Kuwaiti origin with a variable non-syndromic coloboma phenotype (see Table 1) were referred to a pediatric ophthalmologist (K.K.N.) for evaluation of poor vision. The eldest affected child (patient IV:3, Fig. 1A) was 13 years old on first examination and presented with visual acuity of 0.56 LogMAR in the right eye and 1.0 in the left. She exhibited bilateral typical inferior iris coloboma and marked retinochoroidal coloboma (Fig. 1B). In addition, she had a mild lens opacity, divergent squint (exotropia of 35–30 prism diopters), with manifest latent nystagmus and poor fixation in the left eye. On electrodiagnostic testing, pattern visual-evoked potentials (VEPs) were evident to a range of test checks with both eyes open but reduced in amplitude indicating macular pathway dysfunction. The responses from the left eye were degraded compared with the right suggestive of poor vision. Her brother (IV:1) examined initially at 11 years had a visual acuity of 0.54 and 1.0 LogMAR in the right and left eyes, respectively. He exhibited bilateral typical inferior iris coloboma as well as bilateral retinochoroidal and optic disc colobomata. In addition, the crystalline lens was displaced (subluxed) in the right eye. He had a convergent squint (esotropia of 45 prism diopters) and hypertropia in the left eye with rapid manifest latent nystagmus in both eyes. Although electrodiagnostic testing revealed essentially normal flash responses from each eye, monocular pattern VEP testing revealed macular pathway dysfunction affecting the left eye. The youngest of the three siblings (IV:2) aged 9 years at first examination exhibited reduced visual acuities (right eye, 0.16 LogMAR, left eye, hand movements only) and a large retinochoroidal coloboma involving the optic disc in the left eye, whereas the right fundus appeared completely normal (Fig. 1B). In addition, there was a hypertropia and variable esotropia of 20–45 prism diopters, together with manifest latent horizontal, small amplitude pendular nystagmus in the left eye. Normal flash electroretinograms were obtained from both eyes with pattern VEP evident to a range of test checks in the right eye. There was no consistent pattern VEP from the left eye and, in addition, the flash VEP was also degraded in the left eye compared with the right. Together, this is indicative of very rudimentary vision in the left eye, most likely as a result of the optic nerve coloboma. He had evidence of a small corneal diameter in the left eye but was not diagnosed as clinically microphthalmic; refraction was mildly hyperopic, whereas a clinically microphthalmic eye would be expected to exhibit marked hyperopia. Axial length was not formally measured. All three affected children had a full pediatric review and there was no indication for any neurological, developmental or renal abnormalities. Renal function was assessed to be normal by urea and electrolyte function tests; renal ultrasound was not performed, and therefore we cannot exclude the possibility of a structural kidney abnormality. Similarly, neuroimaging was not indicated and therefore not performed on any member of the family. The parents were found to be normal on ophthalmological examination. Unaffected siblings were reported normal after ophthalmological examination and were unavailable for inclusion in the study.

**Table 1.** Summary of ocular phenotype of affected individuals

Patient	IV:1		IV:2		IV:3	
Clinical feature	Left eye	Right eye	Left eye	Right eye	Left eye	Right eye
Globe size	Normal	Normal	Small corneal diameter	Normal	Normal	Normal
Iris	Coloboma	Coloboma	Normal	Normal	Coloboma	Coloboma
Retina	Coloboma	Coloboma	Coloboma involving macula	Normal	Coloboma	Coloboma
Optic disc	Coloboma	Coloboma	Coloboma	Normal	Coloboma	Coloboma
Lens	Normal	Dislocated	Cataract	Normal	Mild cataract	Normal
Electroretinogram	Normal	Normal	Normal	Normal	Normal	Normal
Visual-evoked potential	Poor	Moderate	No response	Good	Poor	Good
Nystagmus	Yes	Yes	Yes	No	Yes	Yes
Squint	Convergent	No	Convergent	No	Divergent	No
Visual acuity (LogMAR)	1.0	0.54	Hand movements only	0.16	1.0	0.56

### Homozygosity mapping and exome sequencing

Assuming an autosomal recessive model of inheritance and in view of the known consanguinity present in the family, we applied an homozygosity mapping strategy to identify the causative genetic mutation underlying the coloboma phenotype. Analysis of genotype data from all three affected individuals to identify regions of extended (>100) consecutive homozygous SNPs revealed two major loci >2 Mb in size common to all three affected individuals: 5.3 Mb on chromosome 8p11.1 delineated by SNPs rs16891730–rs13279327 (chr8g.43106718–48442384; assembly Hg19 NCBI build 37.1) and 15.5 Mb on chromosome 14q11.2 (rs2792135–rs3138056) (chr14g.20409258–35868514; Fig. 1C), in addition to a number of smaller intervals (see Supplementary Material, Table S1). Combined, all of these regions contain ∼320 genes annotated in the RefSeq database.

Given the large number of potential candidate genes, a whole-exome sequencing strategy was adopted to identify the causative mutation. Sequencing of patient IV:1 generated a total of 5.15 Gb of mappable sequence data providing an average depth of 58 reads, yielding a minimum of 5-fold coverage at 90% of the captured exome. Average depth of coverage in all candidate regions of shared homozygosity was sufficient for comprehensive variant detection (29- to 114-fold, Supplementary Material, Table S1) of all exons and for all known annotated genes within all candidate regions regardless of their size. From the aligned reads across the entire captured exome, we identified a total of 20 969 SNP variants of which 8522 were homozygous, plus an additional 991 indels. Variants were filtered by excluding all those present in either dbSNP (build 137) or our local database containing data from 172 exomes with a minor allele frequency >0.1. This identified 26 novel homozygous SNPs and 21 novel indel variants across the entire exome. Of these a single-coding variant mapped to within one of the regions of homozygosity shared by the affected individuals. This variant is situated within the largest homozygous region on chromosome 14 at position g.21993777 (Supplementary Material, Fig. S1) and introduces a premature stop codon within exon 2 (of 2) of the *SALL2* gene (c.85G>T, NM_005407.1) (OMIM 602219). The c.85G>T mutation was confirmed by Sanger sequencing and shown to be homozygous in all three affected siblings and heterozygous in both parents (Fig. 1A). Analysis of DNA from the unaffected siblings (Fig. 1A) would have provided additional genetic evidence supporting pathogenicity of the c.85G>T variant, but was not available for analysis. This variant is also absent from the National Heart Lung and Blood Institute Exome Variant Server database containing sequence data from 6500 exomes (ESP6500, August 2013; http://eversusgs.washington.edu/EVS/). The co-segregation of the homozygous variant with the coloboma phenotype in the three affected children, the unaffected status of the heterozygous carrier parents, the known parental consanguinity and the absence of other pathogenic variants within the shared regions of homozygosity, together with the predicted severity of the mutation led us to conclude that we had identified the pathogenic mutation in this family. This mutation is predicted to produce a severely truncated protein of only 29 residues (p.Glu29*), compared with the 1007 amino acid SALL2 protein, resulting in the loss of translation of 97% of the protein coding sequence including three clusters of zinc-finger motifs shown to be essential for DNA-binding activity (29). Alternatively, this mutation may evoke nonsense-mediated decay mechanisms, which exist to eliminate aberrant mRNA species containing premature truncation codons; however, these mechanisms are not usually activated if the mutation exists in the final exon (30,31). In either of these predicted events, the effect of this mutation is complete loss of any active protein function rendering an essentially null allele. An alternative hypothesis is that a downstream ATG codon could rescue functional domains of the protein resulting in a hypomorphic mutation rather than complete loss of function. However, analysis of mutant mRNA or protein from patient cells to confirm the consequence of the identified *SALL2* mutation was not feasible due to lack of availability of suitable patient tissue.

### Mutation analysis of *SALL2*

To determine the wider potential contribution of *SALL2* mutations to the incidence of coloboma, the coding region of a further 178 patients with coloboma and associated anophthalmia/microphthalmia phenotypes were Sanger sequenced [comprising isolated coloboma (*n* = 49), colobomatous microphthalmia (*n* = 10), syndromic coloboma (*n* = 52), microphthalmia/anophthalmia (*n* = 67)]. This analysis identified three separate previously unreported synonymous coding variants [c.33C>T (p.L11L); c.381C>T (p.V128V); c.315C>T (p.S105S)] in three individuals, each heterozygous for their respective variant, and therefore unlikely to represent pathogenic variation. No other novel potentially pathogenic coding variation was identified in this cohort (Supplementary Material, Table S2).

### Expression analysis of *SALL2*

**Figure 2.**
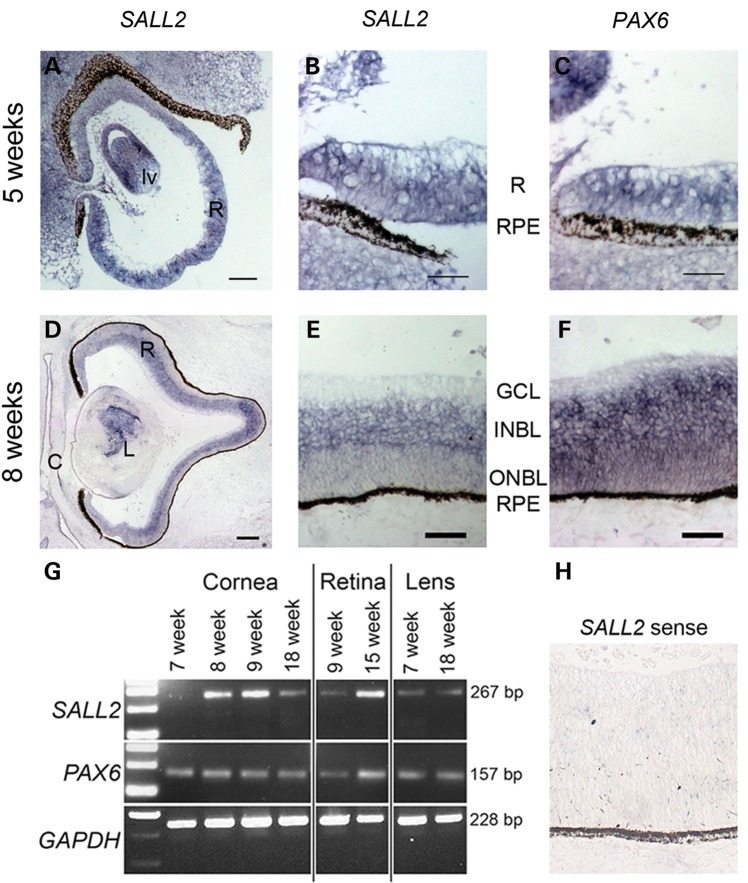
Expression analysis of *SALL2* during human eye development. (**A** and **B**) RNA *in situ* hybridization on sagittal sections showing expression of *SALL2* transcripts throughout the retina (R) and developing lens vesicle (lv) at 5 weeks of development in comparison to *PAX6* expression (**C**). Expression of *SALL2* is maintained at 8 weeks (**D**) but becomes restricted to the inner neuroblastic layer (INBL) as shown magnified in (**E**) as compared with *PAX6* (**F**) which is expressed uniformly throughout the developing neural retina. (**G**) RT–PCR analysis using intron-flanking primers for *SALL2* in total RNA extracted from microdissected cornea, retina and lens tissue from varying stages of development. *PAX6* expression is shown for comparison and *GAPDH* was used as a positive control. (**H**) *SALL2* sense probe shown as a control. GCL, ganglion cell layer;, INBL, inner neuroblastic layer; ONBL, outer neuroblastic layer; RPE, retinal pigmented epithelium. Scale bar represents 50 µm in (B, C, E, F and H), 100 µm in (A), 200 µm in (D).

*SALL2* has previously been reported to be widely expressed in adult human brain as well as the heart, kidney and pancreas (32). In the mouse, expression of *Sall2* has been observed in the developing brain at embryonic day (E) 7.5, becoming restricted to distinct structures within the forebrain, midbrain and hindbrain by E10.5, as well as the developing kidney and neural tube at E11.5 (33,34). Expression has also been observed in the developing lens placode and optic cup at E11.5 consistent with the timing of optic fissure closure in the mouse (E11–E13) (34). To analyse expression of *SALL2* during human eye development, we performed RNA *in situ* hybridization using an antisense riboprobe for *SALL2* on frozen cryosections prepared from human embryonic and fetal eyes. *SALL2* transcripts were detected throughout the retina and developing lens vesicle, in addition to the periocular mesenchyme, at 5 weeks of development (Carnegie stage 15) (Fig. 2A and B), the time at which optic fissure closure commences. Expression was maintained in the developing retina and following completion of fissure closure, at 8 weeks of development, becomes restricted to the inner neuroblastic layer (Fig. 2D and E). Concurrent *PAX6* expression was detected across the neural retina from 5 to 8 weeks of development (Fig. 2C and F). We also confirmed *SALL2* expression in the developing cornea, lens and retina at different developmental stages by reverse transcription PCR analysis of RNA extracted from microdissected human embryonic and fetal ocular tissue together with expression of *PAX6* in these tissues at all time-points examined (Fig. 2G). These data support a role for *SALL2* during human eye development prior to, and maintained during and after, optic fissure closure.

### Histological analysis of *Sall2* null mice

**Figure 3.**
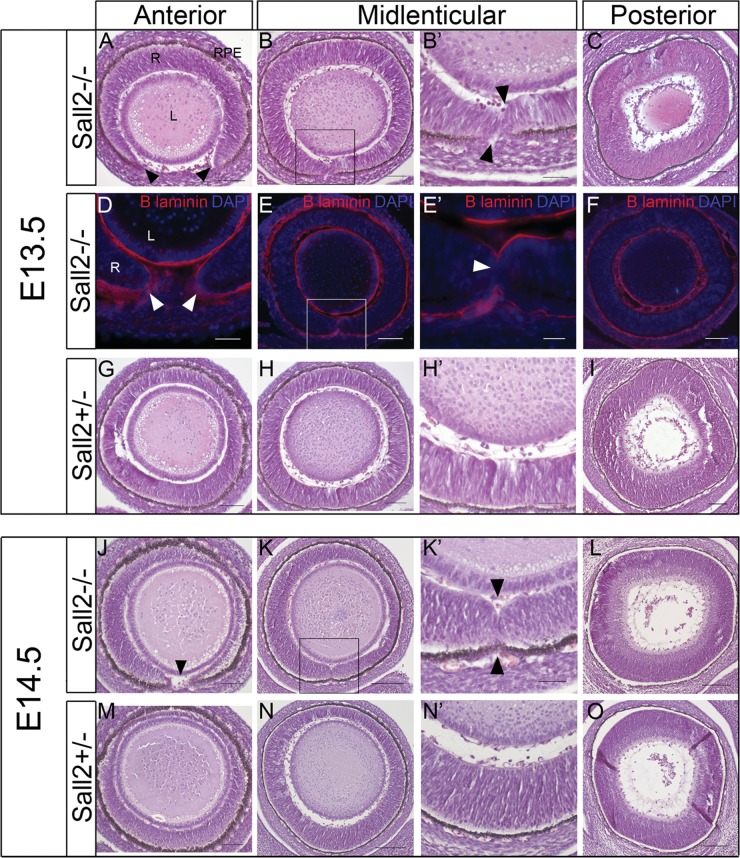
Analysis of optic fissure closure in embryonic eyes from *Sall2^−/−^* mice. (**A**–**C**) Coronal sections of the eye from an E13.5 homozygous *Sall2^−/−^* embryo showing a gap in the neural retina at the anterior aspect of the eye (arrowheads in A). At the mid-lenticular region (B) the retinal margins meet and touch each other with visible indentations at the opposing tissue margins (arrowheads in B′ which shows a magnification of the boxed region in B). Posteriorly (C), the retina appears fused and morphologically continuous and indistinguishable from the control (I). (**D**–**F**) Immunohistochemistry of sections from E13.5 *Sall2^−/−^* eyes using anti-laminin (red) to identify the basement membrane. Laminin staining is observed surrounding the unfused fissure margins anteriorly (arrowheads in D). At the point of contact between the tips of retina in the mid-lenticular region laminin staining is not detectable (E, E′) suggesting dissolution of the basal lamina. Posteriorly, no laminin staining is observed, suggesting the fusion of the optic fissure in this region (F). Cell nuclei are stained with DAPI (shown in blue). (**G**–**I**) Comparative sections from control *Sall2*^+/−^ at E13.5 showing fusion of the optic fissure at all levels of the eye. (**J**–**L**) Coronal sections of the eye from E14.5 *Sall2*^−/−^ embryo showing similar failure of optic fissure fusion in the anterior (arrowhead in J) and mid-lenticular regions (K, K′, arrowheads in K′ indicate indentations in the inner and outer aspects of the retina consistent with incomplete fusion), with a normal appearance posteriorly (L). (**M**–**O**) Representative sections from a control embryo showing the continuous morphological appearance of the fused retina at E14.5. In all images, the ventral aspect is shown at the bottom of the image. L, lens; RPE, retinal pigmented epithelium; R, neural retina. Scale bars represent 50 µm in (B′, E′, H′, K′ and N′), 100 µm in (A, B, D, E, G, H, J, K, M and N) and 200 µm in (C, F, I, L and O).

To further strengthen the evidence supporting the pathogenicity of *SALL2* mutation as a novel cause of coloboma, we investigated loss of *Sall2* function in a mouse model. *Sall2*-deficient mice were previously reported to have no apparent abnormal phenotype when bred on a C57BL/6 genetic background, and low penetrance strain-specific incidence (11–17%) of neural tube defects and significant perinatal lethality when bred onto different mixed genetic backgrounds (33,34). Neural tube closure involves fusion of the neural plate, and bears similarity to the process of optic fissure closure. As previous reports did not analyse the eyes of *Sall2*^−/−^ mice in detail, we bred homozygous *Sall2*^−/−^ mice (on a C57BL/6 background, shown to have 0% penetrance of neural tube defects) for further analysis. *Sall2* null mutant mice showed no overt phenotypic abnormalities. Histological analysis of the eyes revealed a colobomatous phenotype. Table 2 summarizes the analysis of hematoxylin and eosin stained sections of the eye from E13.5 and E14.5 *Sall2*^−/−^ null and *Sall2*^+/−^ heterozygous control embryos, which revealed a penetrant bilateral phenotype in the homozygous *Sall2*^−/−^ embryos. At E13.5, 12 out of 12 (100%) eyes from *Sall2*^−/−^ embryos (from two separate litters) showed a clear gap in the ventral neural retina and retinal pigmented epithelium (RPE) at the anterior aspect of the eye (Fig. 3A). Sequential sections towards the posterior pole showed the unfused optic fissure margins meeting and abutting each other (Fig. 3B and B′). At the mid-lenticular level 6 out of 12 (50%) eyes examined showed evidence of incomplete fusion where both sides of the fissure margins were asymmetric with visible indentations at the inner and outer aspects of the neural retina where the margins meet and a visible gap in the RPE (Fig. 3B and B′). In all *Sall2*^−/−^ eyes examined at E13.5 (100%, *n* = 12) from the mid-lenticular region posteriorly to the developing optic disc, the optic fissure appeared fully fused with an indistinguishable appearance of the dorsal and ventral retina (Fig. 3C), which was morphologically similar to that of control eyes. In contrast to the open fissure observed in the *Sall2*^−/−^ null eyes, sequential coronal sections of *Sall2*^+/−^ control eyes showed complete fusion along the entire length of the fissure (Fig. 3G–I; *n* = 5). No defects were detected in other ocular components.

**Table 2.** Summary of histological analysis of the extent of optic fissure closure in *Sall2^−/−^* null and *Sall2^+/−^* mouse embryos

Stage	Genotype	Anterior		Midline		Posterior		Total eyes
		Open	Closed	Open	Closed	Open	Closed	
E13.5	*Sall2^−/−^*	12 (100%)	0 (0%)	6 (50%)	6 (50%)	0 (0%)	12 (100%)	12
	*Sall2^+/−^*	0 (0%)	5 (100%)	0 (0%)	5 (100%)	0 (0%)	5 (100%)	5
E14.5	*Sall2^−/−^*	9 (100%)	0 (0%)	3 (33%)	6 (67%)	0 (0%)	9 (100%)	9
	*Sall2^+/−^*	0 (0%)	10 (100%)	0 (0%)	10 (100%)	0 (0%)	10 (100%)	10

Similar results were obtained for the histological analysis of eyes at E14.5 with 100% of *Sall2*^−/−^ eyes examined (*n* = 9) showing an open optic fissure in the anterior aspect of the retina, 33% showed evidence of incomplete fusion at the mid-lenticular point and all eyes showed normal morphology in posterior sections compared with controls (Fig. 3J–L). By contrast in all *Sall2*^+/−^ eyes at E14.5 the retina appeared fully continuous with no morphological characteristics of the open optic fissure (Fig. 3M–O; *n* = 10).

During normal optic fissure closure, the neuroepithelial basal lamina at the tips of the fissure margins dissolves in a contact-dependent manner as the tips fuse to form the continuous neural retina and RPE (35). Failure of basement membrane disintegration at the optic fissure margins has been reported as a cause of coloboma in a recent study of Fatty Liver Shionogi mice who present a phenotype similar to human ocular coloboma without microphthalmia, and also in two other coloboma models *Pax2*^−/−^ and *Vax2*^−/−^ mice (36–38). In order to assess whether dissolution of the basal lamina is occurring at the point where the optic fissure margins meet in *Sall2*^−/−^ embryonic eyes, we used an anti-Laminin antibody to identify the basal lamina. Figure 3D shows the basal lamina clearly surrounding the tips of the unfused fissure margins at the anterior aspect of the retina in E13.5 *Sall2*^−/−^ eyes. No laminin staining was observed between the converged tips of the optic fissure at the point of contact in the mid-lenticular region (Fig. 3E and E′), or within the ventral retina in posterior sections where laminin staining was continuous around the lens and outside of the neural retina (Fig. 3F). This indicates dissolution of the basal lamina is occurring correctly in the absence of Sall2 when the fissure margins make contact, consistent with the complete fusion of the optic fissure observed from the midline to the posterior pole of the eye.

**Figure 4.**
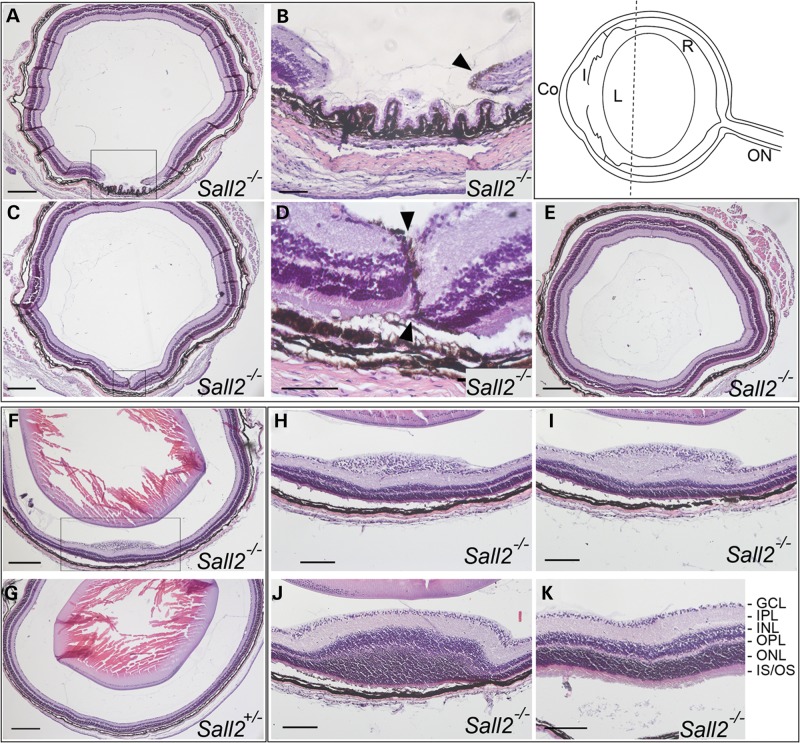
Histological analysis of eyes dissected from P20 *Sall2^−/−^* mice. Top right. Schematic representation of a mouse eye showing the plane of sectioning and approximate anterior location of the observed retinal abnormality (dotted line). Co, cornea; I, iris; L, lens; R, neural retina; ON, optic nerve. (**A**–**E**) Coronal sections of the eye from a P20 homozygous *Sall2*^−/−^ mouse showing a gap in the neural retina visible anteriorly (A and B). In serial sections towards the posterior pole, the tips of the retinal margins meet with residual pigment visible surrounding the tips of the margins (arrowheads in B and D which show magnifications of boxed region in A and C, respectively). (E) More posteriorly, beyond the mid-lenticular region, the retina appears fused and indistinguishable from normal. Note, the lens was removed during processing. (**F**) Representative coronal section from a homozygous *Sall2*^−/−^ mouse showing localized elevated lesion of the retina as compared with (**G**) equivalent coronal section of eye from P20 heterozygous *Sall2*^+/−^ mouse showing no abnormality. (**H**–**J**) Consecutive anterior-to-posterior sections showing magnification of boxed region in (F) showing progressive swelling and disorganization of ganglion cell layer (H), inner plexiform layer (I) and inner and outer nuclear layers (M). In more posterior sections, the retina shows normal lamination (**K**). GCL, ganglion cell layer; IPL, inner plexiform layer; INL, inner nuclear layer; OPL, outer plexiform layer; ONL, outer nuclear layer; IS/OS, photoreceptor inner and outer segments. Scale bars represent 200 µm in (A, C, F, G and E), 50 µm in (B and D) and 100 µm in (H–K).

Given the observation that the optic fissure appeared to fuse normally at the back of the eye, we next performed analysis of eyes from postnatal day (P) 20 *Sall2*^−/−^ mice (from three separate litters, *n* = 18 eyes) to determine if the anterior colobomatous defect persists, or whether the delayed embryonic fusion process is ameliorated at later stages. This analysis identified two distinct classes of retinal abnormality in homozygous mutant animals. Six of 18 eyes (33%) displayed a retinal coloboma phenotype similar to that seen in the embryo with a gap in the peripheral ventral neural retina behind the ciliary body (Fig. 4A and B; Supplementary Material, Fig. S2). Sequential sections towards the posterior pole showed the unfused margins meeting and abutting each other (Fig. 4C and D). During normal development, the presumptive retinal pigmented epithelial (RPE) layer has been reported to invert into the merging fissure margins prior to fusion and eventual RPE separation from the neural retina (35). In the *Sall2*^−/−^ retina a residual layer of pigment was observed at P20 between the two opposing tips of the neural retina (arrowhead, Fig. 4B and D). In this case, although retinal lamination appeared histologically normal at the juxtaposed margins, the layers were mis-aligned and visible indentations were apparent at the inner and outer aspects of the neural retina where the margins meet, consistent with incomplete fusion (Fig. 4D, arrowheads). Other regions where the retinal margins failed to make contact showed evidence of disorganization of the retinal lamina (Supplementary Material, Fig. S2, arrowheads). Seven other *Sall2*^−/−^ mutant eyes showed a single focal anteriorly localized lesion within the neural retina (Fig. 4F, H and I), appearing in some cases as if the outer layer on the one side had traversed the inner layer on the other side (see Fig. 4I). Outside of the lesion, the histology was indistinguishable from that of wild-type and heterozygous eyes (Fig. 4K). Within the elevated lesion, which protruded into the vitreal space, the ganglion cell layer, and the inner and outer nuclear layers were abnormally thickened (Fig. 4I and J), whereas the plexiform layers appeared disorganized with an increased inner plexiform layer and reduced outer plexiform layer. In all eyes examined, the retina of homozygous *Sall2*-null mice was indistinguishable from wild type from the mid-lenticular region to the posterior pole where the optic nerve exits the eye (Fig. 4E and G) consistent with the conclusion that the retinal abnormalities in the *Sall2* null mice are restricted to the anterior aspect of the ventral retina. Comparative analysis of both eyes from the same animal showed cases of bilateral coloboma (Supplementary Material, Fig. S2), bilateral retinal lesion, as well as unilateral coloboma with contralateral retinal lesion. The remainder of the homozygous *Sall2*^−/−^ eyes examined histologically at P20 (*n* = 5), as well as heterozygous *Sall2*^+/−^ mice of equivalent age (*n* = 4) showed no visible morphological abnormality. There was no evidence for a small eye/microphthalmic phenotype in any of the homozygous *Sall2*^−/−^ mutant eyes at either the embryonic or adult stages investigated. Together, these data show that loss of *Sall2* causes coloboma in mice as well as humans, and suggests that *Sall2* is required for correct growth of the optic fissure margins towards each other and their alignment, rather than the process of dissolving the basement membrane.

### Analysis of PAX2 and PAX6 in relation to *SALL2* expression

**Figure 5.**
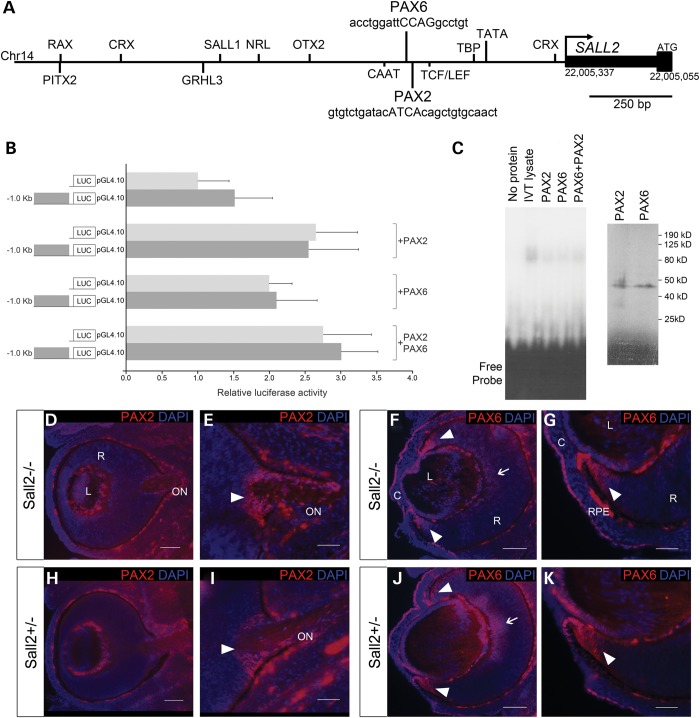
Analysis of SALL2 in relation to PAX2 and PAX6. (**A**) Schematic diagram of 1.6 kb of the human *SALL2* proximal promoter region showing selected transcription factor-binding sites relevant to ocular development. (**B**) Luciferase reporter assay in HEK293 cells showing that neither PAX2 nor PAX6 activate transcription of a luciferase reporter construct (LUC) containing 1023 bp of the *SALL2* promoter encompassing putative binding sites for both proteins. No significant difference in activation was observed between the *SALL2* promoter construct (dark gray bars) compared with the promoterless reporter (pGL4.10, light gray bars) when co-transfected with PAX2 or PAX6 expression constructs either singly or in combination. Data are presented as mean ± SD of three repeat experiments each performed in triplicate. (**C**) EMSA showing that no specific protein:DNA complex is formed between *in vitro* translated PAX2 or PAX6, either singly or in combination, and a DNA probe containing both predicted binding sites in the *SALL2* promoter as shown in (A). Right hand panel shows *in vitro* translation of PAX2 and PAX6 proteins incorporating [^35^S]-l-methionine showing equivalent amounts of protein are synthesized in each reaction; bands of the expected sizes of 44 and 46 kDa are observed for PAX2 and PAX6, respectively (**D**–**G**) Immunohistochemistry of sagittal sections of the eye from E13.5 mouse embryos stained with anti-PAX2 (red). Specific expression of PAX2 is observed in the developing optic nerve head (arrowheads in E and G). No difference in expression pattern is observed between *Sall2*^−/−^ (D and E) and control *Sall2*^+/−^ (F and G) eyes. (**H** and **I**) Immunohistochemical analysis of PAX6 (red) in sagittal eye sections from E13.5 *Sall2*^−/−^ (H and I) and *Sall2*^+/−^ control (**J** and **K**) embryos. PAX6 expression is detected in the retina (arrows) with strong expression seen in the anterior periphery of the retina (arrowheads), as well as the corneal epithelium and lens. No difference in PAX6 protein expression was observed between *Sall2*^−/−^ and control eyes. Nuclei were counterstained with DAPI (blue). C, cornea; L, lens; R, retina; ON, optic nerve. Scale bars represent 50 µm in (E, G, I and K) and 100 µm in (D, F, H and J).

Finally in order to identify coloboma genes that might be regulating *SALL2* in the eye during optic fissure closure, we performed an *in silico* analysis of putative transcription factor-binding sites in ∼1.6 kb of the *SALL2* proximal promoter (chr14:g.22005337–22007000). Matinspector (www.genomatix.de) analysis identified a number of potential ocular transcription factor-binding sites in this region (Fig. 5A), including adjacent predicted binding sites for PAX6 (chr14:g.22005800–22005818) and PAX2 (chr14:g.22005773–22005801), two transcription factors implicated in human coloboma pathology, located ∼430–460 bp upstream of the start of transcription of *SALL2* (Fig. 5A). Comparative genomic analysis of the human and mouse *Sall2* promoters showed limited overall sequence conservation; however, Matinspector also identified a number of putative PAX2- and PAX6-binding sites in the murine *Sall2* proximal promoter region (Supplementary Material, Fig. S3). Alteration of *PAX2* function is a common cause of coloboma and studies in mouse and zebrafish models have highlighted its function in morphogenetic events required for optic fissure closure (28,36,39,40). To test whether PAX2 and/or PAX6 are capable of regulating expression of *SALL2 in vitro*, we cloned a 1.0 kb region of the proximal promoter upstream of a luciferase reporter gene. Co-transfection of PAX2 or PAX6 expression constructs separately or in combination did not elicit a significant activation of the *SALL2* promoter when compared with the promoterless reporter vector (Fig. 5B). Furthermore, electrophoretic mobility shifts assays did not provide evidence in support of specific binding of either PAX2 or PAX6 (or in combination) to the *SALL2* promoter (Fig. 5C). To assess the opposite model of *Pax2*/*Pax6* expression being altered by loss of *Sall2* during fusion, we analyzed the distribution of PAX6 and PAX2 proteins in *Sall2*^−/−^ embryonic eyes by immunohistochemistry. By E13.5 the downregulation of PAX2 within the ventral optic cup has occurred normally in mutant embryos; PAX2 was detected only in a wedge of cells surrounding the optic nerve head as previously described (36,41). PAX6 was detected within the retina and lens. These expression patterns were unaltered in *Sall2*^−/−^ embryos compared with controls (Fig. 5D–K). *Pax2* mutant eyes show persistence of the basal lamina when the converging tips make contact, as judged by the abnormal presence of laminin across the retina (36). Here, the normal regulation and distribution of PAX2 observed in the Sall2^−/−^ eye is consistent with the observed dissolution of the basement lamina in the posterior hemisphere and the normal formation of the optic nerve. These data together suggest that SALL2 is not directly regulated by PAX6/PAX2 and may be either genetically downstream of, but not directly regulated by PAX2/PAX6 binding to the proximal promoter, or alternatively *SALL2* may function in an independent pathway required for correct alignment of the fissure margins.

## DISCUSSION

### SALL family genes and human disease

Our study identifies *SALL2* as the third member of the *spalt*-like family of transcription factor genes to cause a human birth defect. SALL/Spalt family genes are widely studied in model organisms and play a crucial role during the development of a number of systems (42,43). The *SALL2* gene contains a zinc-finger domain homologous to the *Drosophila* region-specific homeotic gene *spalt* (*sal*) (42). There are four human *spalt-*like genes each encoding an N-terminal C2HC type zinc finger, in addition to three (SALL2, SALL4) or four (SALL1, SALL3) clusters of C2H2 class zinc-finger domains required for interaction with DNA and other proteins (42,44). Phylogenetic analysis identifies conserved orthologues of *SALL1*, *SALL3* and *SALL4* genes throughout most metazoa, whereas *SALL2* appears to have evolved following the divergence of the mammalian lineage as orthologous genes have not been identified in non-mammalian organisms (45). Previously, we reported *SALL1* mutations cause the developmental disorder Townes-Brocks syndrome (OMIM 107480) and *SALL4* mutations cause Okihiro (Duane-Radial Ray; OMIM 607323) syndrome (46,47). These syndromes exhibit dominant inheritance, whereas we identify homozygous loss of *SALL2* as the cause of a recessive eye condition. Similar to the mutation we have identified in *SALL2* (p.Glu29*), premature termination mutations lacking all paralogous zinc-finger domains have been reported for both *SALL1* and *SALL4* resulting in loss of function of the mutant proteins (46–48). A recessive hypomorphic mutation in *SALL1* has also been reported in association with a variant Townes-Brocks phenotype (49) implying that dosage of these genes is critical during embryogenesis. In the case of *SALL2*, we find that a single gene copy is sufficient for normal ocular development, as demonstrated by the lack of an observable phenotype in the unaffected parents and heterozygous *Sall2* mutant mice.

Functional redundancy between *Sall* factors is likely to compensate for the heterozygous loss of *Sall2* in the eye (33,34,50), particularly *Sall1* which is expressed in the developing eye and can be associated with retinochoroidal coloboma in some cases of Townes-Brocks syndrome (48,51). Similarly, redundancy is likely to restrict the phenotype to the eye, despite the wider expression pattern of the *Sall2* gene; for example, homozygous deletion of *Sall1* results in severe kidney dysgenesis, whereas *Sall2*-deficient mice have normal kidneys despite both genes being expressed during kidney development (33,52).

### Variable penetrance of *SALL2*/*Sall2*-related phenotype

The variable penetrance of the coloboma phenotype observed in the adult *Sall2*^−/−^ mice accords both with the variable penetrance of neural tube defects previously observed on different genetic backgrounds (34) and the variable expressivity and incomplete penetrance in other examples of both murine and human coloboma (37,38). However, at embryonic stages the observed coloboma phenotype was fully penetrant and bilateral in all embryos investigated, indicating some degree of amelioration at later stages of development when a third of postnatal animals exhibited no apparent abnormality. The phenotype is also variable within the pedigree we describe, as demonstrated by individual IV:2 who had a normal fundus appearance in one eye, indicating that the optic fissure closure defect can be overcome in the absence of SALL2 by the effects of other environmental, stochastic or genetic factors. Implicated environmental risk factors include vitamin A deficiency during gestation consistent with the role of retinoic acid (RA) signaling in early eye patterning (53). Variability in the initiation site or timing of optic fissure closure, both between and within species, may explain the different presentation and location of the defect. In mice initiation of closure of the optic fissure has been reported to occur at a posterior level close to the developing optic disc rather than from a central midpoint in a proportion of animals (35). Whereas in human development fusion initiates in the center of the fissure and progresses both anteriorly towards the rim of the optic cup and posteriorly towards the optic stalk (54). Genetic variation influencing the expression levels of, as yet unidentified, targets of SALL2 or their downstream effectors is also likely to contribute to phenotypic variability in both species.

### Human genes required for optic fissure fusion

Correct optic fissure closure requires six crucial steps: (i) early morphogenesis of the optic vesicle, (ii) growth and patterning across the dorso-ventral axis of the bilayered optic cup to form an incomplete globe, (iii) alignment and contact between the neuroepithelia on either side of the fissure, (iv) adhesion of the fissure margins, (v) dissolution of the basement membrane and (vi) fusion and separation of the neural retina and RPE to form a complete globe. Comprehensive identification of the genes involved in these steps, their function and interactions may offer a basis for future therapeutic interventions, but there is much to be learned in this respect. From previous human genetic studies and complementary analysis in animal models, a number of genes have been implicated in different stages of this process. For instance, midline Sonic hedgehog signaling is required for (i) and loss of *SHH* signaling causes holoprosencephaly, which in milder forms includes coloboma and microphthalmia (18). Both RA and bone morphogenetic protein (BMP) signaling play key roles in (ii) and disruption of these pathways can cause coloboma associated with microphthalmia and anophthalmia [*RBP4* (OMIM 180250), *STRA6*, *ALDH1A3*, *BMP4* (OMIM 112262), *GDF6*, *GDF3* and *SMOC1* mutations disrupt RA and BMP signaling, respectively] (13,20–22,55–59), whereas the transcription factors *RAX*, *PAX6* (OMIM 607108), *VSX2*, *OTX2* (OMIM 600037), *SIX3* (OMIM 603714) and *SOX2* (OMIM 184429) are involved in eye field specification and neural progenitor proliferation (i–ii) and loss of function causes anophthalmia/microphthalmia, including rare reports of coloboma (16,17,23,25,60,61). Mutations in *C12orf57* have recently been identified in patients with colobomatous microphthalmia associated with developmental delay, seizures and corpus callosum abnormalities suggesting a role at similarly early levels of development (62). Analysis of *PAX2*, a cause of optic disc and retinal coloboma (OMIM 120330) (28), has identified roles in both patterning the optic stalk/neural retina boundary together with *PAX6* (i), and for fissure closure and correct development of the optic disc (v and vi) (63). In addition, a genetic signaling cascade for coloboma centered around *PAX2* has been elucidated involving SHH and BMP4 signaling as upstream regulators (39,64). Although coloboma is a common association with microphthalmia, this does not necessarily mean that coloboma is a non-specific outcome of microphthalmia. A recent study of 141 patients with microphthalmia, anophthalmia and coloboma phenotypes revealed a similar number of microphthalmic cases without coloboma (24%) to those with optic fissure closure defects and normal globe size (23%) (65). None of the patients in the present study were clinically diagnosed as microphthalmic. Furthermore, analysis of homozygous mutant *Sall2*^−/−^ eyes at embryonic and adult stages failed to show any evidence of a small eye or microphthalmia phenotype. These observations combined with the localized nature of the retinal coloboma phenotype identified suggest that *SALL2* mutation plays a specific role in disruption of the closure process at steps (iii) and (iv), but not (v).

### Genetic pathways and mechanisms implicated in optic fissure closure

Where does *SALL2* fit within the molecular pathways important for fissure closure? Our analysis suggested that *SALL2* plays a specific role in optic fissure closure required independently of *PAX2*. Prompted by the presence of putative PAX2-binding sites in the *SALL2* promoter we investigated the idea that *SALL2* is genetically downstream of, and potentially directly regulated by, PAX2. No evidence was found supporting PAX2 regulation of the *SALL2* promoter. Our findings also refuted the alternative model of coloboma caused by disruption of PAX2 function by showing normal localization of PAX2 in the cells encircling the optic disc and normal dissolution of the basal lamina, which requires PAX2, in the *Sall2*-deficient eyes. PAX2 deficiency primarily causes a posterior optic disc defect in mice (63), whereas in the *Sall2*^−/−^ mice normal closure of the posterior globe was observed. This finding is also consistent with a previous observation that *Pax2* expression is unaltered in the developing kidney of *Sall2*-deficient mice (33). Interestingly, a JNK1/2>BMP4/SHH>PAX2 signaling pathway is implicated in both optic fissure and neural tube closure defects (39). *Jnk1^−/−^ Jnk2^−/+^* mice (lacking activity of members of the Jun N-terminal Kinase, JNK, group of mitogen activated kinases) exhibit coloboma associated with loss of Shh and Bmp4 signaling, and reduced levels of Pax2, while addition of Bmp4 restored Shh and Pax2 expression (39). The JNK1/2>BMP4/SHH pathway may also be important for regulating *SALL2* expression, independently of PAX2. In *Sall2^−/−^/Sall4^+/−^* mouse embryos that develop exencephaly, like JNK1/JNK2/PAX2-deficient embryos, increased levels of apoptotic cells and failure of elevation and fusion of the neural folds were reported (34). Similarities in the molecular pathways and mechanisms of these different tissue fusion processes are likely to emerge from future analysis. Other recent studies have provided new evidence for the association between dysregulation of cell death and coloboma in zebrafish (40,66), though it is not yet clear how similar these fusion processes are to those in mammals.

What is the role of SALL2 in the eye? The molecular function of the SALL2 protein is largely unknown, although a role as a tumor suppressor has been suggested (67). Previous *in vitro* studies demonstrated that SALL2 exhibits growth arrest and pro-apoptotic properties and interacts directly with the p75 neurotrophin receptor, which is implicated in the control of programmed cell death during retinal development (68–70). Perturbation of cell death pathways, and/or promotion of cell proliferation (68,71), could plausibly explain both the open fissure, and the appearance of an abnormal retinal mass in the *Sall2* null mice. Future studies to investigate the expression pattern of other coloboma-associated genes, and the identification of the downstream targets and effectors of SALL2 function during embryonic development in *Sall2*^−/−^ mice will help to further elucidate essential genetic pathways and their role in coloboma formation.

## Summary

In summary, using a combined homozygosity mapping and exome sequencing approach, we have identified a novel homozygous mutation in *SALL2* in three siblings variably affected with retinochoroidal coloboma. This mutation (c.85G>T) is predicted to result in a truncated protein of only 29 amino acids lacking all important functional domains of SALL2. We have shown that *SALL2* is expressed in the developing human retina during the period of optic fissure closure and that *Sall2*-deficient mice exhibit an ocular coloboma phenotype providing a new model to investigate developmental closure and fusion mechanisms. In *Sall2*-deficient embryos the fissure margins fail to meet in the anterior portion of the globe, whereas in the posterior region closure is completed as normal dissolution of the basal lamina and fusion occurs. This is the first report of a critical role for *SALL2* in eye morphogenesis in both humans and mice, and the gene should therefore be considered for mutation screening in patients with coloboma.

## MATERIALS AND METHODS

### Ethical approval

Informed written consent was obtained from the parents prior to collection of blood samples, DNA extraction and analysis. The study was approved by the UCL Institute of Child Health/Great Ormond Street Hospital for Children Joint Research Ethics Committee. All murine experiments were carried out using protocols reviewed and approved under license by the United Kingdom Home Office under the Animal (scientific procedures) Act 1986.

### Homozygosity mapping and exome sequencing

DNA samples from all three affected individuals were genotyped using the Human610-Quad BeadChip (Illumina) scanned using an iScan system and data analyzed using Beadstudio v3.2 (Illumina) to identify regions of consecutive homozygous SNPs (>100) common to all affected individuals. Whole-exome sequencing was performed to identify candidate genetic variants. Three micrograms of genomic DNA from patient IV:1 was subject to in-solution enrichment of exonic sequences using the SureSelect Human All Exon Kit v.1 (Agilent) according to the manufacturer’s instructions. Adaptors were ligated for sequencing using a 76 bp paired-end run on an Illumina Genome Analyser IIx platform. Reads were aligned to the human reference genome (Hg19 NCBI build 37.1) using Novoalign software (version 2.07.04; Novocraft) (http://www.novocraft.com/main/index.php). Variant calling (SNPs and indels) was performed using SAMtools (version 0.1.7) together with Dindel (version1.01) (http://www.sanger.ac.uk/resources/software/dindel/) using default values and a minimum SNP and indel quality of 30 and minimum SNP and gap mapping quality of 50.

### *In situ* hybridization

Human embryonic and fetal eyes were fixed in 4% (w/v) phosphate-buffered formaldehyde solution (PFA) and equilibrated in 30% sucrose solution for cryoprotection prior to freezing in an optimal cutting temperature (OCT) compound (RA Lamb). Sections were cut on a Leica CM1900 UV cryostat to 14 μm thickness and collected on Superfrost-Plus glass slides (VWR). *In situ* hybridization was performed for 18 h in hybridization buffer containing digoxigenin-incorporated riboprobes. Riboprobes were generated from an 840 bp fragment of the 3′ untranslated region of human *SALL2* (NM_005407.1) amplified using primers 5′-GACCTTGGTAGAGGAGCTGA-3′ and 5′-AGGAATGCCACATACTGGTT-3′ and cloned into pGEMT-Easy (Promega). The template for the *PAX6* probe has been described elsewhere (72). Slides were incubated with anti-digoxigenin conjugated with alkaline phosphatase (Roche) diluted 1:1000 in 2% sheep serum. Expression patterns were visualized with a Nitro-Blue Tetrazolium Chloride/5-Bromo-4-Chloro-3′-Indolyphosphate p-Toluidine Salt (NBT/BCIP) system (Roche). Sections were mounted in DPX-mounting medium (RA Lamb) and viewed on a Zeiss Axioplan 2 and images were captured with a Jenoptik C14 digital camera (OpenLab, Improvision). *In situ* data are from the analysis of five human eyes, tested in at least three-independent experiments for each antisense probe; sense probes were used as controls and gave no hybridization signal.

### Immunohistochemistry

OCT compound was removed from cryosections by washing for 15 min in phosphate-buffered saline (PBS) at 37°C. Sections were then blocked with 10% (v/v) goat serum, 1% (w/v) bovine serum albumin in PBS containing 0.1% (v/v) Triton X-100 for 1 h at room temperature preceding primary antibody incubation. Primary antibodies were incubated in blocking solution for 2 h at room temperature at the following concentrations: PAX6 (Covance) 1:300, PAX2 (Lifespan Biosciences) 1:100, and Laminin (Abcam) 1:200; Triton X-100 was excluded from the blocking buffer for incubations with anti-Laminin. After several washes with PBS, sections were incubated with Goat anti-rabbit AlexaFluor594 (Life Technologies), 1:800 dilution, in blocking solution at room temperature for one hour, followed by a 1:3000 dilution of the nuclear dye 4′,6-diamidino-2-phenylindole, dihydrochloride (DAPI; Life Technologies) for 10 min at room temperature. Slides were mounted following several washes in PBS with Citifluor AF-1 mountant (Electron Microscopy Science).

### Reverse transcription–PCR

RNA was extracted from microdissected human fetal cornea, retina and lens using Trizol reagent (Invitrogen) according to the manufacturer's protocol. First-strand cDNA synthesis was performed with 50 ng total RNA via M-MLV Reverse Transcriptase (Promega) and random hexamer oligonucleotide primers. Second strand synthesis was performed with gene-specific primers from exon 1 to exon 2 (flanking intron 1) for *SALL2*, 5′-AACCCCAACAGTTAATCTCG-3′ and 5′-TATGCTCTGTGTCCATGACC-3′, and exon 9 to exon 10 for *PAX6*, 5′-CACCAGTGTCTACCAACCAA-3′ and 5′-TTGCATAGGCAGGTTATTTG-3′.

### Histological analysis of *Sall2* mutant mice

*Sall2* knockout mice were maintained on a C57BL/6 background and genotyped by PCR of genomic DNA as described previously (34). Experimental litters were generated by overnight mating, with the day of finding a copulation plug designated as E0.5. Litters were dissected from the uterus at Days 13.5 and 14.5 in PBS. Whole heads were dissected from embryonic mice and individual eyes dissected from P20 mice following labeling of the dorsal edge with Tissue Marking Dye (Cancer Diagnostics) to orientate the eye, fixed in 4% PFA, dehydrated through a graded alcohol series and chloroform and embedded in paraffin. Parasagittal sections through the head cut the optic cup en face and were performed to allow visualization of the optic fissure as a gap in the ventral retina. Microtome sections were cut at 8 µm mounted on Superfrost-Plus slides and rehydrated through ethanol series prior to staining with hematoxylin and eosin, followed by subsequent dehydration in ethanol, clearing in Histo-clear (National diagnostics) and mounting.

### Plasmid constructs

Full-length *PAX2* (NM_003987) and *PAX6* (IMAGE Consortium Clone ID 3880468) (73) cDNA clones in mammalian expression vectors were purchased from Cambridge Bioscience and Source Bioscience UK, respectively. PCR primers were designed to amplify a 1023 bp region of the *SALL2* proximal promoter (Chr14:g.22005060–22006082) as follows: SALL2-1023F 5′-GTGCTCAGCCTAATTTTTGTG-3′ and SALL2-1023R 5′-GGGTAGAGAGTTGGGAGAGG-3′. PCR products were generated from human genomic DNA, purified and cloned directly upstream of the luciferase gene in pGL4.10 (Promega).

### Electrophoretic mobility shift assay

PAX2 and PAX6 proteins were synthesized using the TnT T7 and SP6 Coupled Reticulocyte Lysate Systems (Promega). EMSAs were performed using following radiolabeled DNA probe encompassing both the PAX2- and PAX6-binding sites in the proximal promoter of SALL2, double stranded by annealing to the complimentary oligonucleotide: 5′-GATCACCTGGATTCCAGGCCTGTGTCTGATACATCACAGCTGTGCAACTT-3′. Ten microliters of *in vitro* translated protein were added to 50 pmol of probe in a total 40 µl binding reaction containing 7.5 mm Tris, 37.5 mm KCl, 3.25% glycerol, 30 µg bovine serum albumin, 75 µm dithiothreitol, 750 µm ethylenediaminetetraacetic acid. Binding reactions were incubated at room temperature for 20 min prior to electrophoresis through an 8% non-denaturing polyacrylamide gel.

### Cell culture and transient transfection

Human embryonic kidney 293 cells were maintained in Dulbecco's modified Eagle medium supplemented with 10% fetal bovine serum at 37°C in humidified air containing 5% CO_2_. Transient transfection assays were performed using Lipofectamine 2000 reagent (Life Technologies) following the manufacturer's instructions. Briefly, 2.5 × 10^4^ cells/well were seeded into a 96-well tissue culture plate. Cells were transfected with 20 ng of luciferase reporter and 30 ng of each expression construct either individually or in combination as indicated in Figure 5. pRLSV40 (Promega) was cotransfected in all experiments to control for transfection efficiency, and the total amount of transfected DNA was normalized to 120 ng/well by the addition of pBluescript. Cells were harvested 24 h following transfection and assayed for luciferase activity using the Dual-Luciferase Reporter Assay System (Promega), using a BMG Fluostar Optima microplate reader. Luciferase activity for each expression vector was determined by normalization to the level of baseline luciferase activity of the promoterless pGL4.10 reporter. Data are presented as mean ± SD from three-independent experiments each performed in triplicate. Two-way Student's *t*-test was used to determine significance between means.

### Mutation analysis

PCR primer pairs were designed to amplify the coding sequence of *SALL2* (NM_005407.1) using Primer3 (http://frodo.wi.mit.edu/). Primer sequences were as follows: 1, forward 5′-TCTGCTTCACAGTGATTTGC-3′, reverse 5′-TGCATCTCAACTCCTTCAAA-3′; 1A, forward 5′-TTTCTCACTCCAGCTTCTCC-3′, reverse 5′-CCTACGCAGAGAATCATGC-3′; 2.1, forward 5′-GGTTACTGGCCTCCTTGTTA-3′, reverse 5′-GTCGATTCTGGAGGTAATGG-3′; 2.2, forward 5′-CAGAGAGGAGAGGAGAGGAGT-3′, reverse 5′-GTGGTAAAGGTGGAAGAAGG-3′; 2.3, forward 5′-AAGACACTGGCATCTTCCTC-3′, reverse 5′-AAACGGTTTCCACAGACATT-3′; 2.4, forward 5′-CCAAAGTATTTGGCAGTGAC-3′, reverse 5′-TACTTTCTGCCACTCCACTG-3′; 2.5, forward 5′-TTTGTGCTCATGAAAGCAGT-3′, reverse 5′-AGATTACCCCTGGTGGAGA-3′; 2.6, forward 5′-CGAGTGCTTAGCTGTCCTC-3′, reverse 5′-TCTTCTGAATCACCTCTCACTG-3′; 2.7, forward 5′-AGGATGAGGAAGAAGAGGAA-3′, reverse 5′-GTCTTCTGATGCTCCTCCAG-3′; 2.8, forward 5′-GACCTTGGTAGAGGAGCTGA-3′, reverse 5′-ATCAGGGCTCATAACTCTGG-3′. PCR products treated with Shrimp Alkaline Phosphatase (GE Healthcare) and Exonuclease I (New England Biolabs) were directly sequenced using BigDye Terminator v1.1 sequencing chemistry (Applied Biosystems) using a 3730XL DNA Analysis System (Applied Biosystems). Sequences were analyzed using Sequencher v4.8 software (Gene Codes Corp.) and compared with the reference sequence for *SALL2* (NM_005407.1).

## SUPPLEMENTARY MATERIAL

Supplementary Material is available at *HMG* online.

## FUNDING

This research was supported by the Ulverscroft Foundation, the Child Health Research Appeal Trust, Action Medical Research, the University of London Central Research Fund, the Rosetrees Trust and the National Institute for Health Research (NIHR) Biomedical Research Centre (BRC) at Great Ormond Street Hospital for Children NHS Foundation Trust (GOSH) and University College London (UCL). J.C.S. is supported by the Great Ormond Street Hospital Children's Charity. P.L.B. is supported by the Wellcome Trust. J.L. is supported by the Medical Research Council UK. L.I. was supported by an Action Medical Research Training Fellowship. GOSgene at the UCL Institute of Child Health is supported by the NIHR BRC at GOSH and UCL. The human embryonic and fetal material were provided by the Joint MRC UK (G0700089)/Wellcome Trust (GR082557) Human Developmental Biology Resource (http://hdbr.org). Funding to pay the Open Access publication charges for this article was provided by UCL.

## Supplementary Material

Supplementary Data
