# Supplementary material for: Mutation of SALL2 causes recessive ocular coloboma in humans and mice
Source: Hum Mol Genet. 2014 Jan 9;23(10):2511–26. doi: 10.1093/hmg/ddt643 (PMC3990155; doi:10.1093/hmg/ddt643)
Supplement: Supplementary Data [file supp_23_10_2511__index.html]

Mutation of SALL2 causes recessive ocular coloboma in humans and mice — Mutation of SALL2 causes recessive ocular coloboma in humans and mice — Mutation of SALL2 causes recessive ocular coloboma in humans and mice — Supplementary Data 

# Mutation of *SALL2* causes recessive ocular coloboma in humans and mice

## Supplementary Data

Supplementary Data

**Files in this Data Supplement:**

- Supplementary Data - Docx file
