# Supplementary material for: Mutation of SALL2 causes recessive ocular coloboma in humans and mice
Source: Hum Mol Genet. 2014 Jan 9;23(10):2511–26. doi: 10.1093/hmg/ddt643 (PMC3990155; doi:10.1093/hmg/ddt643)
Supplement: Supplementary Data [file supp_ddt643_ddt643supp.docx]

**Supplementary Figure S1. Homozygous mutation in patient IV:1 identified by exome sequencing.**

**
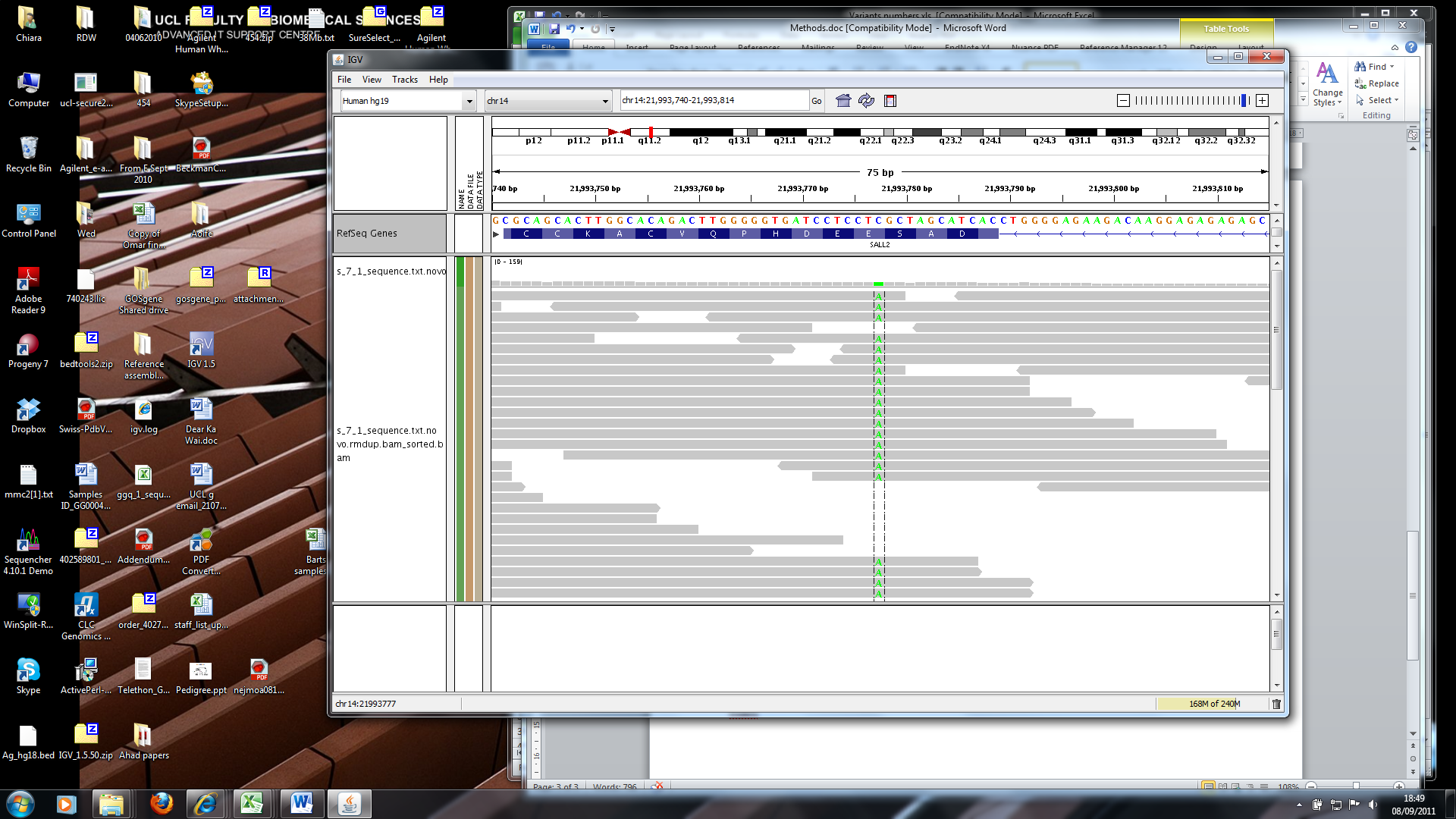
**

Individual overlapping sequencing reads from patient IV:1 visualized by the Integrative Genomics Viewer visualization tools (Broad Institute) showing 21 fold depth of coverage of the mutation in exon 2 of the *SALL2* main transcript (NM_005407.1) highlighted in green (base pair position g.14:21993777C>A, Hg19 NCBI build 37.1).

**Supplementary Figure S2. Histological sections of eyes from P20 *Sall2*^-/-^ mice showing retinal coloboma.**

**
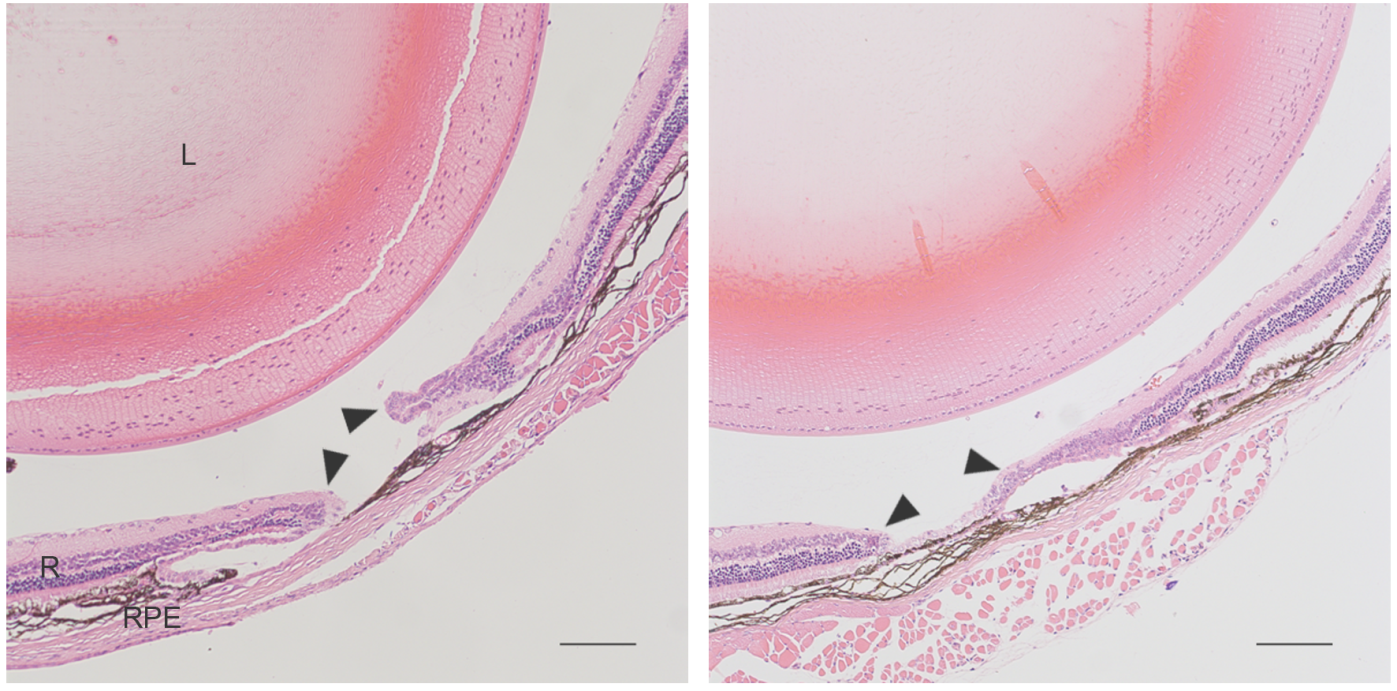
**

Coronal sections of left and right eyes from a P20 homozygous *Sall2*^-/-^ mouse showing a gap in the ventral retina at the anterior aspect of the eye. Arrowheads indicate the opposing unfused tips of the retina. L, lens, R, retina, RPE, retinal pigmented epithelium. Scale bar 300 µm.

**Supplementary Figure S3. Comparative genomic analysis between 1.6 Kb of the human *SALL2* and murine *Sall2* promoters.**

**
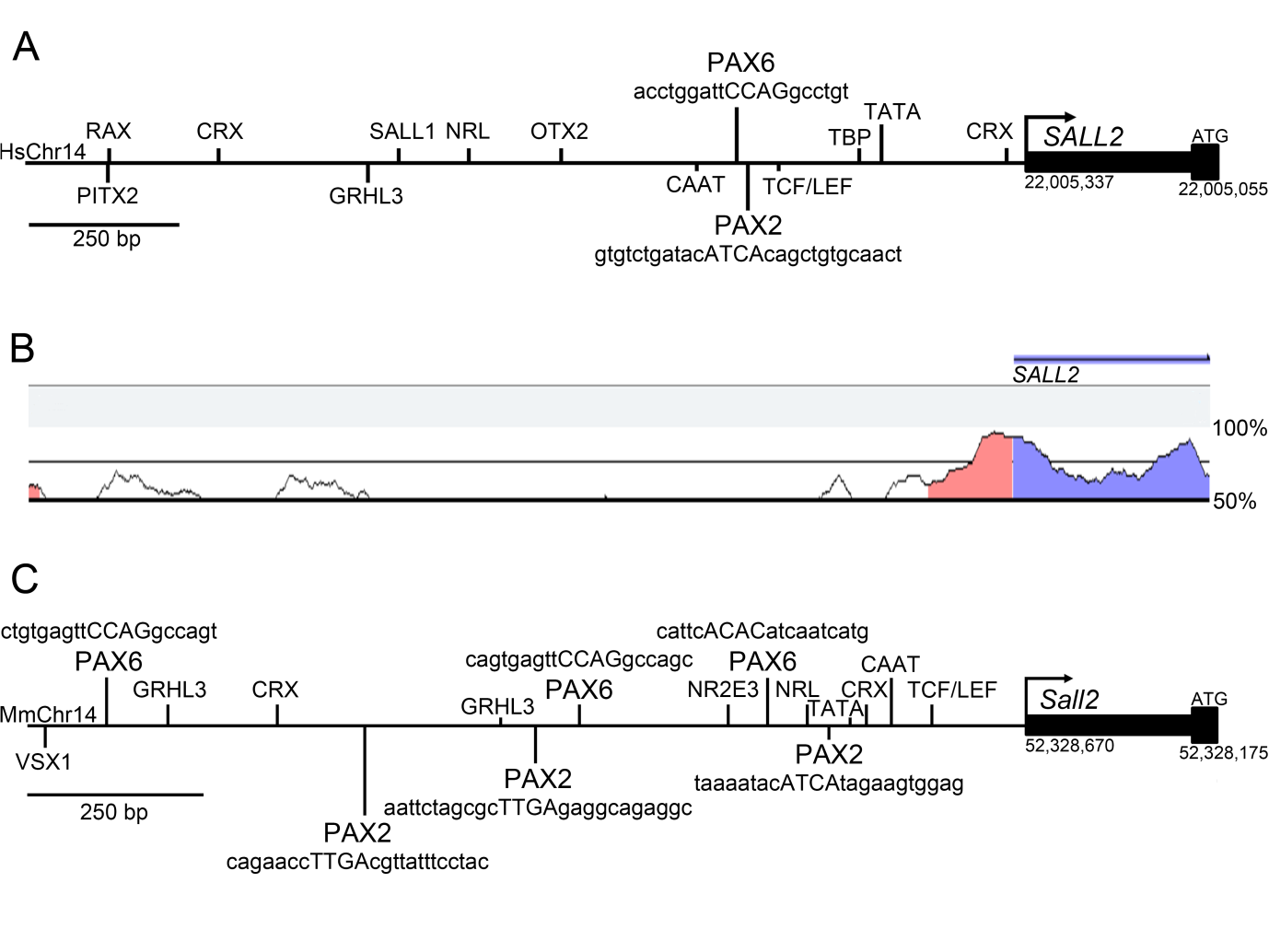
**

A) Schematic diagram of 1.6 Kb of the human *SALL2* proximal promoter region showing selected transcription factor binding sites relevant to ocular development identified using Matinspector (www.genomatix.de). B) Comparative sequence alignment of comparable regions between the human and mouse promoters generated using VISTA (genome.lbl.gov/vista/index.shtml) showing percentage sequence identity between the two regions on the y axis. The pink highlighted region indicates greater than 75% sequence identity, the blue highlighted region indicates the start of exon 1 of the *SALL2* gene up to the initiation ATG codon. C) Schematic showing relevant ocular transcription factor binding sites present in the equivalent murine Sall2 proximal promoter region. Co-ordinates given relate to the Hg19 and Mm10 builds of the human and mouse genomes respectively.**Supplementary Table S1. Summary of regions of shared homozygosity identified between affected individuals >1 Mb.**

| **Chr** | **Region**  **(Hg19 build GRCh37)** | **Size (bp)** | **Flanking SNPs** | **Exome sequencing median coverage** |
| --- | --- | --- | --- | --- |
| 1 | 195,915,909 – 197,867,662 | 1,951,753 | rs7537893-rs1092326 | 93 |
| 2 | 1,199,920 – 2,234,892 | 1,034,972 | rs4461289-rs17338414 | 33 |
| 4 | 127,530,731 – 129,221,901 | 1,691,170 | rs6840819-rs6534677 | 85 |
| 6 | 27,261,324 – 28,716,345 | 1,455,021 | rs7746199-rs1233585 | 77 |
| 8 | 43,106,718 – 48,442,384 | 5,335,666 | rs16891730-rs13279327 | 63 |
| 13 | 57,405,169 – 58,832,013 | 1,426,844 | rs9597450-rs7992943 | 29 |
| 14 | 20,404,091 – 35,890,861 | 15,486,770 | rs2792135-rs3138056 | 45 |
| 14 | 41,823,890 – 43,136,625 | 1,312,735 | rs8011757-rs1616139 | 114 |
| 14 | 47,236,264 – 48,479,196 | 1,242,932 | rs724701-rs12883308 | 105 |
| 15 | 41,249,273 – 42,270,730 | 1,021,457 | rs745299-rs4924592 | 30 |

**Supplementary Table S2. *SALL2* variants identified in cohort of 178 patients with coloboma and anophthalmia/microphthalmia phenotypes.**

| **Position** | **cDNA position**  **(NM_005407.1)** | **Function** | **Amino acid** | **Observed allele freq.** | **dbSNP rs ID** |
| --- | --- | --- | --- | --- | --- |
| g.14:22004922 | c.73+61C>T | intronic | none | C=0.997  T=0.003 | novel |
| g.14:21994229 | c.33C>T | synonymous | p.L11L | C=0.997  T=0.003 | novel |
| g.14:21993638 | c.224C>G | non-synonymous | p.S75C | C=0.943  G=0.057 | rs2242527 |
| g.14:21993547 | c.315C>T | synonymous | p.S105S | C=0.997  T=0.003 | novel |
| g.14:21993498 | c.364C>T | non-synonymous | p.P122S | C=0.854  T=0.146 | rs1263811 |
| g.14:21993481 | c.381C>T | synonymous | p.V128V | C=0.997  T=0.003 | novel |
| g.14:21993363 | c.499C>A | non-synonymous | p.P167T | C=0.997  A=0.003 | rs150787846 |
| g.14:21993359 | c.503C>T | non-synonymous | p.P168L | C=0.997  T=0.003 | rs144885457 |
| g.14:21992122 | c.1740G>A | synonymous | p.G580G | C=0.980  A=0.020 | rs45442799 |
| g.14:21991630 | c.2232G>A | synonymous | p.G744G | G=0.977  A=0.023 | rs61746515 |
| g.14:21991626 | c.2236G>C | non-synonymous | p.G746R | G=0.670  C=0.330 | rs1263810 |
| g.14:21991589 | c.2273C>G | non-synonymous | p.P758A | C=0.972  G=0.028 | EVS |
| g.14:21990897 | c.2965T>C | non-synonymous | p.S989P | T=0.997  C=0.003 | rs142138768 |
| g.14:21990796 | c.*42G>C | 3’UTR | none | G=0.989  C=0.011 | rs45458492 |

‘novel’ defined as not present in dbSNP or the National Heart, Lung and Blood Institute Exome Sequencing Project Exome Variant Server database (http://evs.gs.washington.edu/EVS/). ‘EVS’ indicates variant detected in the Exome Variant Server but not present in dbSNP.
